# Preliminary Investigations on Intradiscal Pressures during Daily Activities: An In Vivo Study Using the Merino Sheep

**DOI:** 10.1371/journal.pone.0069610

**Published:** 2013-07-24

**Authors:** Sandra Reitmaier, Hendrik Schmidt, Renate Ihler, Tugrul Kocak, Nicolas Graf, Anita Ignatius, Hans-Joachim Wilke

**Affiliations:** 1 Institute of Orthopedic Research and Biomechanics, Center of Musculoskeletal Research, University of Ulm, Ulm, Germany; 2 Department of Orthopedic Surgery, University of Ulm, Ulm, Germany; Van Andel Institute, United States of America

## Abstract

**Purpose:**

Currently, no studies exist, which attest the suitability of the ovine intervertebral disc as a biomechanical *in vivo* model for preclinical tests of new therapeutic strategies of the human disc. By measuring the intradiscal pressure *in vivo*, the current study attempts to characterize an essential biomechanical parameter to provide a more comprehensive physiological understanding of the ovine intervertebral disc.

**Methods:**

Intradiscal pressure (IDP) was measured for 24 hours within the discs L2-L3 and L4-L5 via a piezo-resistive pressure sensor in one merino sheep. The data were divided into an activity and a recovery phase and the corresponding average pressures for both phases were determined. Additionally, IDPs for different *static* and *dynamic activities* were analyzed and juxtaposed to human data published previously. After sacrificing the sheep, the forces corresponding to the measured IDPs were examined *ex vivo* in an axial compression test.

**Results:**

The temporal patterns of IDP where pressure decreased during activity and increased during rest were comparable between humans and sheep. However, large differences were observed for different *dynamic activities* such as standing up or walking. Here, IDPs averaged 3.73 MPa and 1.60 MPa respectively, approximately two to four times higher in the ovine disc compared to human. These IDPs correspond to lower *ex vivo* derived axial compressive forces for the ovine disc in comparison to the human disc. For activity and rest, average ovine forces were 130 N and 58 N, compared to human forces of 400-600 N and 100 N, respectively.

**Conclusions:**

*In vivo* IDPs were found to be higher in the ovine than in the human disc. In contrast, axial forces derived *ex vivo* were markedly lower in comparison to humans. Both should be considered in future preclinical tests of intradiscal therapies using the sheep. The techniques used in the current study may serve as a protocol for measuring IDP in a variety of large animal models.

## Introduction

Disc degeneration is considered to be one of the most important causes of chronic low back pain [1] and it is associated with structural changes including a gradual degradation of proteoglycans and a progressive loss of water content and intervertebral disc height [2]. During the past 20 years, a better understanding of disc function and technical advancements in the field of tissue engineering has resulted in numerous strategies to replace or regenerate different compartments of the disc after herniation or in early stages of painful disc degeneration [3,4]. Based on this concept, (a-) cellular scaffolds are planned for implantation to induce a differentiated *in situ* regeneration of the structurally altered main disc compartments, the nucleus pulposus and the annulus fibrosus.

Before any of these strategies can be introduced into the clinic, extensive preclinical testing is required to demonstrate both the safety and efficacy of the new therapeutic option. Large animal models in particular are important for examining the suitability and practicality of many surgical procedures. Since the early 1990s the sheep has been increasingly used as an animal model for intervertebral disc research [5–12]. Early *in vivo* studies justified the usage of sheep by the structural similarities to humans in their particular scope of application. Comparative biomechanical and anatomical studies [13,14] provided scientific evidence for comparability between humans and sheep. When biological parameters, e.g. cellular and biochemical composition of the disc, were also found to be similar between both species [15,16], it was concluded that the sheep is justified as an *in vivo* model for regenerative strategies of the disc [17,18].

Biomechanically, however, it remains a debate on principles as to whether to use or not to use animal models for spinal research at all [19]. At first glance, because of the upright posture of humans, quadrupeds do not appear to be suitable as representative *in vivo* models for spinal applications in general and consequentially for regenerative strategies of the disc in particular. Valid *in vivo* data, however, are still lacking. For example, the intradiscal pressure (IDP), which is an essential biomechanical parameter for the cellular differentiation of the disc [20–23], has never been investigated in sheep *in vivo* and compared to humans.

The objective of the current study therefore was to measure the IDP over 24 hours *in vivo* to provide a more comprehensive physiological understanding of the ovine intervertebral disc. To evaluate any possible dependency of IDP from the spinal level, two intervertebral discs, L2-L3 and L4-L5, were investigated. The sheep IDP measurements will be compared to published data on humans [24–26]. The data will assist with the scientific interpretation of past and future sheep studies and will be an important step towards the closer characterization of the sheep as a model for disc regeneration strategies. In addition, the techniques used in the current study may serve as a protocol for measuring intervertebral disc pressure in other large animal models.

## Materials and Methods

### Experimental design

The IDP in the discs of the L2-L3 and L4-L5 spinal motion segments were measured in one female merino sheep (~3 years, 90 kg) over 24 hours with the animal performing different kinds of activities. For comparative purposes, these ovine *in vivo* IDPs for different activities were juxtaposed to human data collected by Nachemson and Elfstrom, 1970, Wilke et al., 1999 and Sato et al., 1999. After sacrificing the animal, compression tests on the same motion segments were performed to estimate the axial compressive force that corresponds to the *in vivo* measured pressure values for standing and lying.

### Ethics statement

Permission for the animal experiment was granted by the local ethical committee (Regierungspräsidium Tübingen, Reg. -Nr. 1032). The proper housing, feeding and care, as well as all interventions relating to the animal welfare were carried out in strict compliance with the stipulations of FELASA (Federation of European Laboratory Animal Science Associations) and conform to ARRIVE guidelines (Animal Research: Reporting of In Vivo Experiments) [27]. The animal was slowly familiarized to the researchers, the animal care staff, as well as the measuring device prior to the surgical interventions. The animal was allowed to move freely without external constraints.

### Animal experiment – surgical intervention

While still in its stall, the animal was sedated with an intramuscular injection of Rompun^®^ 2% (xylazine hydrochloride, 0.2 mg/kg body wt, Bayer, Leverkusen, Germany). For perioperative care an analgesic (Rimadyl^®^, carprofen, 4.0 mg/kg body wt, Pfizer, Karlsruhe, Germany) and an antibiotic (Veyxyl^®^, amoxicillin trihydrate, 7.0 mg/kg body wt, VeyxPharma GmbH, Schwarzenborn, Germany) were injected subcutaneously. Following pre-medication and transportation to the operating room, the animal was anesthetized by the application of an intravenous bolus of thiopental (Thiopental Inresa 0.5 g, 5.0 mg/kg body wt, Inresa GmbH, Freiburg, Germany). For the subsequent duration of the surgery an inhalational anesthesia was administered using isoflurane (Forene^®^, Abbott GmbH, Wiesbaden, Germany).

**Figure 1:**
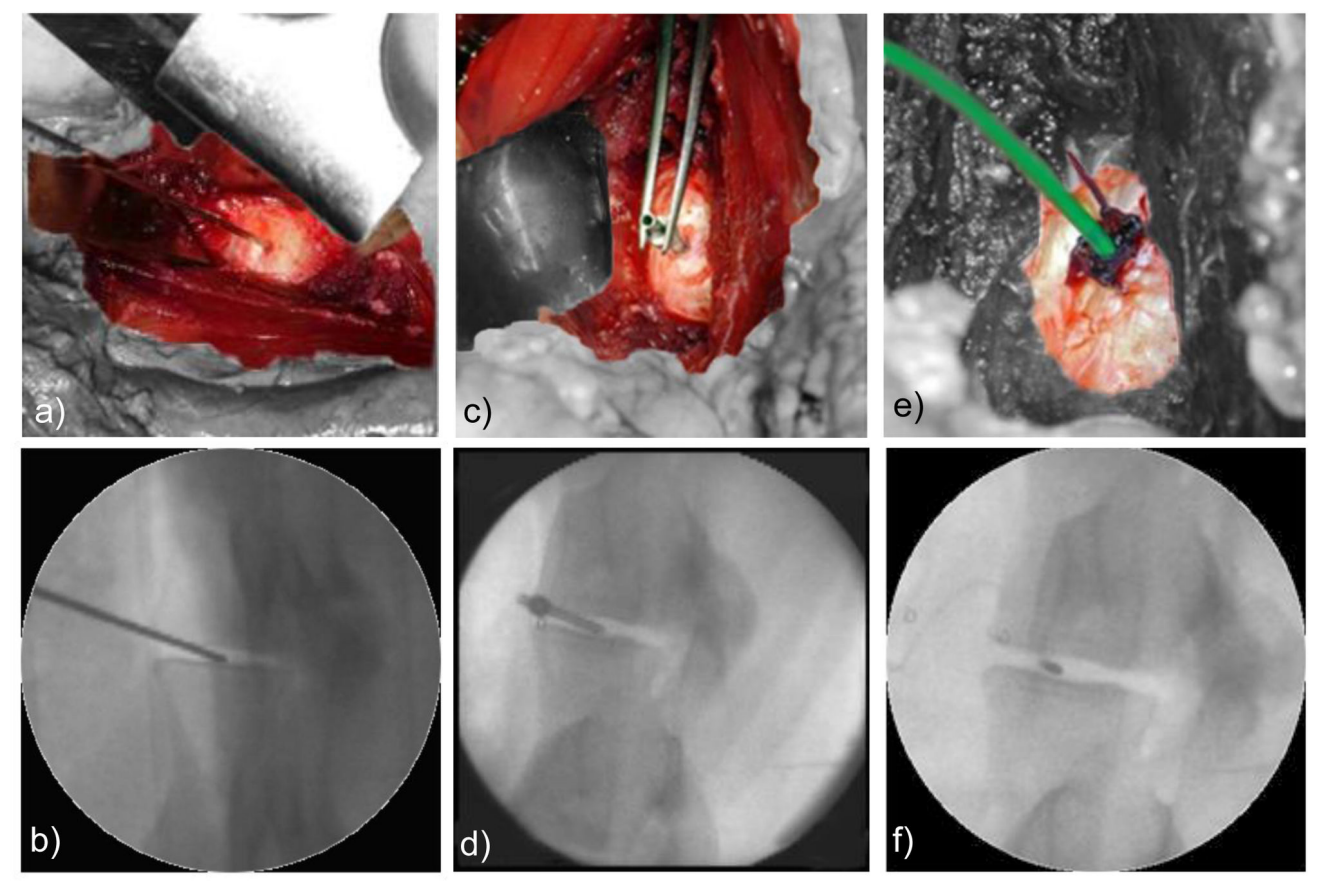
Implantation of the pressure sensor. Surgical site (upper row) and radiographic view (lower row, 60° oblique) of the sensor positioning. a, b) Puncture of the intervertebral disc L2-L3 with a wire. c, d) Positioning of the custom-made hollow canula to introduce the sensor into the ovine disc and e, f) implanted sensor in the center of the nucleus pulposus.

In accordance with measurements on humans, the IDP within the spinal level L4-L5 was measured. To additionally determine whether IDP depends on the lumbar level, the IDP in the segment L2-L3 was also recorded. To allow for a multisegmental access to the lumbar spine, a retroperitoneal approach was performed with the animal lying on its right side [28]. Via blunt dissection of the abdominal muscles, the retroperitoneal cavity was reached and pursued in the direction of the lumbar spine, avoiding any damage to the peritoneum. To expose the intervertebral discs, the psoas muscle was split. Subsequently, the left lateral side of the discs L2-L3 and L4-L5 was punctured with a wire (Ø1.45 mm, non-cutting taper tip, Figure 1 a,b) over which a custom-made cannula (inner Ø1.45 mm, Figure 1 c,d) was inserted into the nucleus pulposus. After removal of the wire, a pressure sensor (Ø 1.45 mm, FMSPEZ50, Mammendorfer Institut für Physik und Medizin GmbH, Mammendorf, Germany) was inserted through the cannula into the center of the nucleus under radiographic control (Figure 1e,f). Keeping the tip of the pressure sensor in place, the cannula was removed from the disc to the proximal end of the silicone cable and out of the abdomen of the sheep. At the entry opening, the cable was sutured to the outer layers of the annulus. To avoid gathering of annular tissue by continuous suture, e.g. purse-string suture, three modified interrupted sutures were used. For each, the thread end was firstly looped around the cable and knotted. The thread was not cut at this stage, but the atraumatic needle was guided through the superficial layers of the annulus right next to the entry opening and strictly underneath the first knot. Finally, the thread end was knotted with the needle end so that both knots tightly attached to each other and onto the annular surface. This guaranteed that the sensor was not dislodged from the disc. The abdominal wall was subsequently sutured layer by layer with regard to the position of the sensor. After surgery, the animals were transported to the stall for recovery.

**Figure 2:**
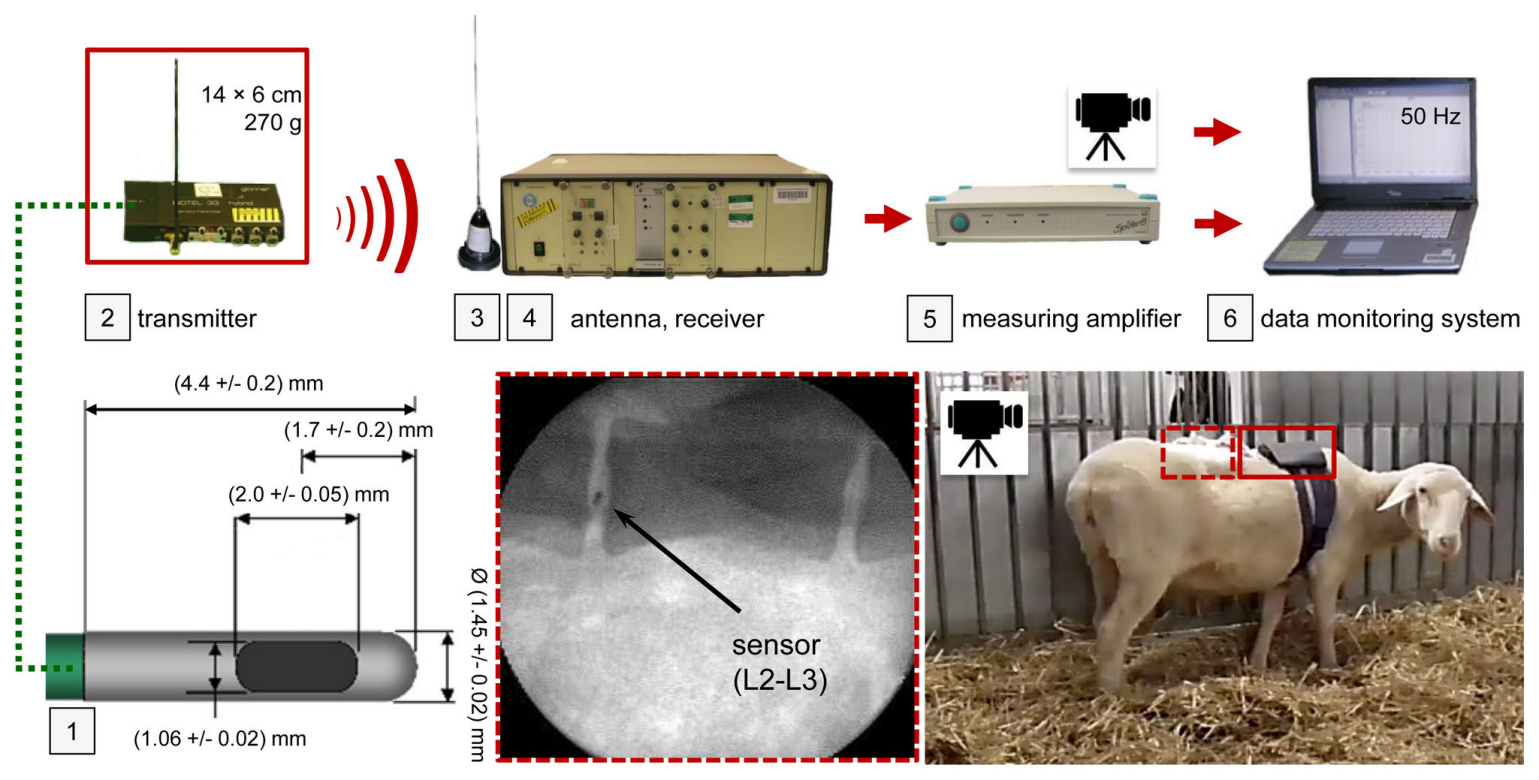
Methology of the in vivo experiment. Activities of the sheep were monitored by video and the experimental staff. The two identical pressure sensors [1] were connected to a telemetry transmitter [2] carried by the experimental animal inside a backpack. The signal was transferred wirelessly to the receiver, passed a measuring amplifier and was collected together with the footage in a data monitoring system at a rate of 50 Hz.

Pressure data were transmitted using a telemetry system (BIOTEL 33 hybrid, Glonner Electronic GmbH, Martinsried, Germany, Figure 2). The transmitter (14 × 6 cm, mass: 270 g) was affixed to the animal inside a backpack without causing any restriction to mobility. The output signal was recorded at 50 Hz via a measurement amplifier (DMCplus; HBM Hottinger Baldwin Messtechnik, Darmstadt, Germany). After sacrifice, the position of the pressure sensors was verified radiographically.

### Animal experiment - data analyses

The total test period lasted 24 hours and started directly after the sensors were sutured to the annulus. In contrast to humans, sheep do not have a continuous sleeping phase at night or continuous daily activity without interruptions. To summarize the mean IDP during *rest* and *active phases*, a simple division of data into night and day was not appropriate for sheep. Periods in which the animal was lying asleep or snoozing (even throughout the course of day) were classified as *rest phases*, and periods in which the animal was standing or walking around were classified as *active phases*.

The IDP was evaluated for seven different activities: 1) lying intraoperatively, 2) lying postoperatively, 3) sleeping, 4) standing, 5) lying down, 6) standing up from a lying position, and 7) walking. Activities 1 through 4 are *static* while activities 5 through 7 are *dynamic*. For the *static activities*, a mean value of the recorded IDP values was calculated over a time window of approximately 10 sec. For the *dynamic activities*, the maximum occurring IDP value was evaluated. The results for the seven different activities are given as median and ranges, each based on IDP values for the specific activity at six different time points. Except for lying intraoperatively (activity 1) and lying postoperatively (activity 2), the time points were randomly assigned over the whole experimental period of 24 hours. Lying intra- and postoperatively, however, were calculated from six equally distributed IDPs within the equivalent time frame of surgery and the animal’s recovery directly postoperatively, respectively.

Statistics were performed using Gnu R [29]. Calculated differences in IDP between the ovine spinal levels investigated were tested using the paired, two-sample Wilcoxon signed rank test to a significance level of p<0.05.

### Laboratory experiment – specimen preparation

After sacrificing the animal, the lumbar spine was removed without causing any damage to the spinal structures. The motion segments in which the pressure sensors were inserted *in vivo* were extracted and carefully stripped of muscles and soft tissues, preserving the structural integrity of the discs, the facet joints and ligaments. During preparation, the sensor remained in the position in which it was implanted during surgery. The discs were horizontally aligned and the upper third of the cranial and the lower third of the caudal vertebra were embedded in polymethylmethacrylate (PMMA, Technovit 3040, Heraeus Kulzer, Werheim, Germany).

### Laboratory experiment – compression test

The two motion segments L2-L3 and L4-L5 were fixed into an electro-mechanical material testing machine (Z010, Zwick GmbH & Co. KG, Ulm, Germany). Specimens were preloaded at 20 N for 15 minutes in order to minimize a potential excessive postmortem hydration caused by elimination of muscle forces. Subsequently, specimens were subjected to three loading cycles with a maximum axial compression force of 500 N at a loading rate of 30 N/s. While the first two cycles served as preconditioning, the final cycle was used for data analyses. During the compression test, the nucleus pressure was recorded at 50 Hz.

## Results

### Animal welfare

Neither intra-nor postoperative health-related complications occurred. The animal recovered from anesthesia without any problems, began to eat immediately upon awakening and displayed an unimpaired gait with a species-typical behavior approximately 2 hours after surgery. There was no obvious sign of constricted mobility of the sheep due to the measuring unit.

### Animal experiment

**Figure 3:**
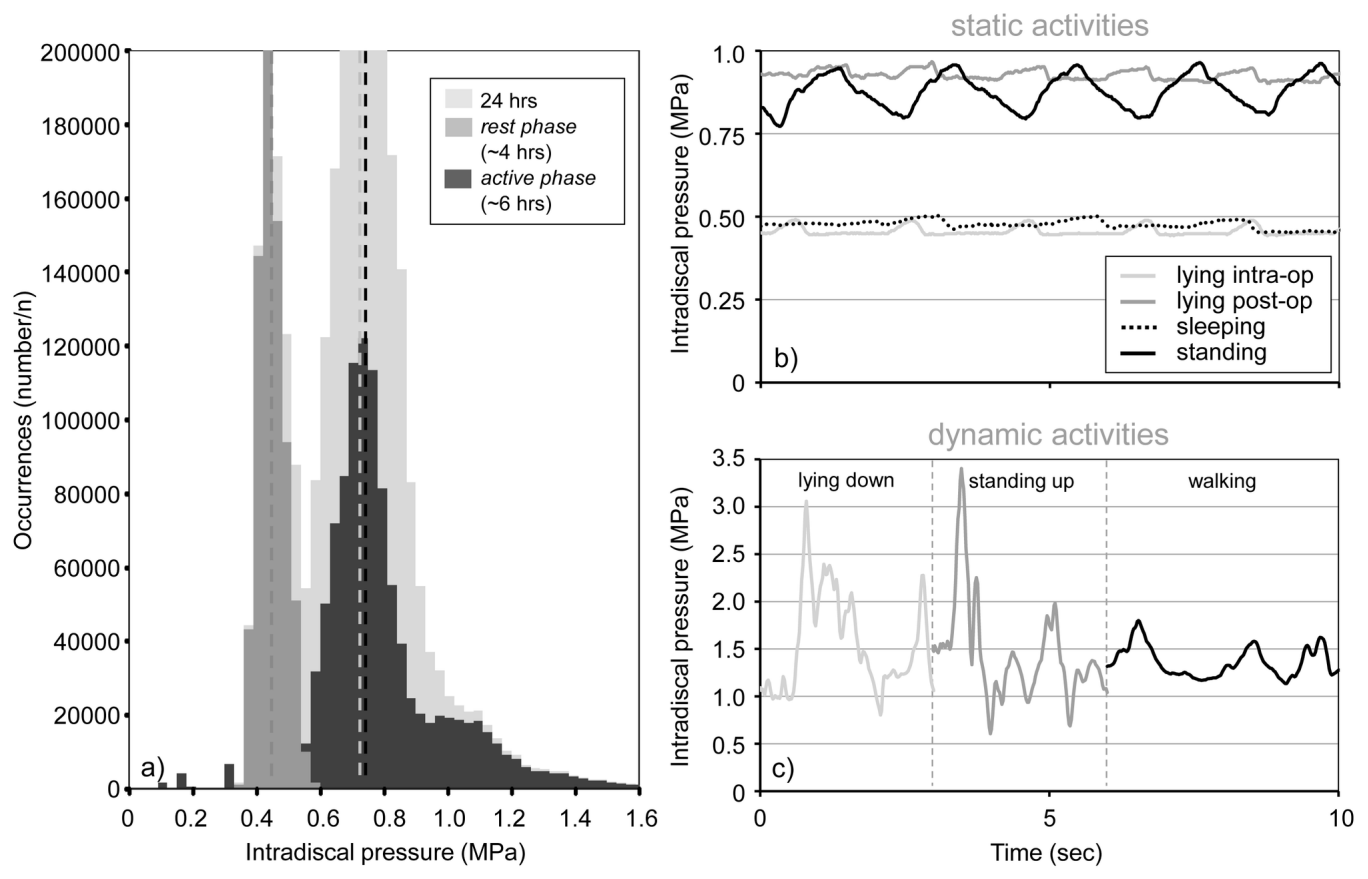
24-hours-proportion of ovine IDPs and representative pressure profiles. a) Frequency distribution of the total data set of IDPs throughout the 24 hour test period subdivided into *active phases* and *rest* and 10-seconds real-time profiles of the a) four different *static* and b) three different *dynamic activities*.

The histogram of the total pressure set in its entirety was not normally distributed, but followed a bimodal pattern with a significant polarization into two frequency distributions and the occurrence of two local maxima (Figure 3a). The mean pressure for the *rest phase* was 0.5 MPa, while the mean pressure for the *active phase* was 0.75 MPa. Both phases, with their mean values and the unimodal distribution, were in good agreement with the corresponding modal values of the histogram of the entire data set. They were both similar in shape and approximately symmetrical.

Figure 3b and c summarize the IDPs for different *static and dynamic activities* for both disc levels over 10 sec. The IDP oscillated with time due to the breathing rate of the sheep. The differences in IDP between inspiration and expiration were shallower during surgery (~0.08 MPa) than during different activities (~0.15 MPa), reflecting the influence of narcotics on respiration. For standing, the highest IDP value of ~1.24 MPa was measured directly after surgery when the disc was probably fully rehydrated through fluid imbibition. As standing after surgery was not representative for standing in general, this value was not integrated in the results for standing, but remained separate. For lying, the IDP was with ~0.43 MPa lowest during surgery and at ~0.73 MPa highest directly after surgery.

**Figure 4:**
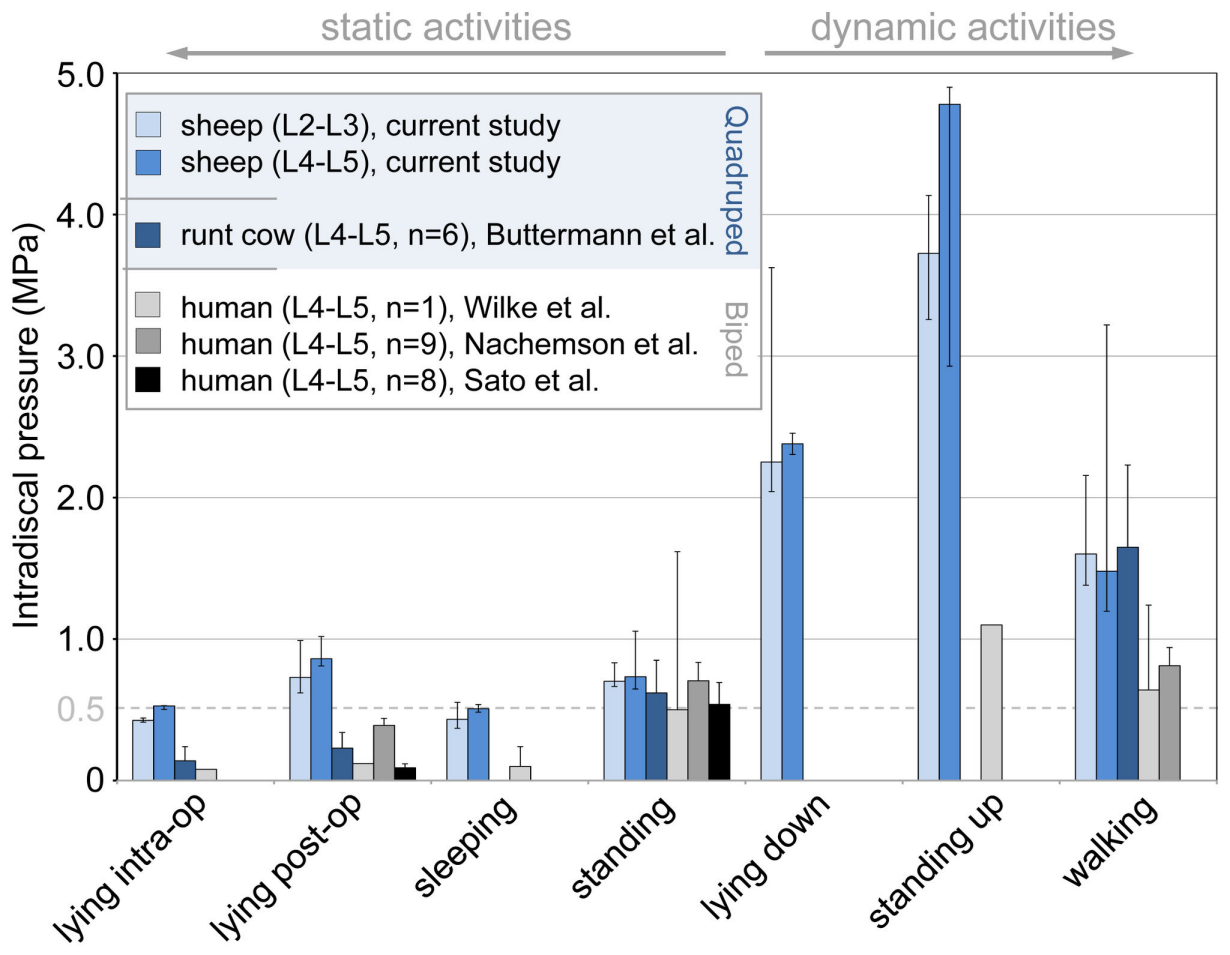
Ovine *in vivo* pressure values in comparison to literature. Intradiscal pressure in L2-L3 and L4-L5 for various static and dynamic activities measured in one sheep. Ovine values represent the median and ranges for six different time points throughout the test duration in comparison to both quadrupedal [30] and human measurements [24–26].

Except for lying intraoperatively, only negligible differences in IDPs were seen for the two disc levels (Figure 4). In the following, therefore, only the IDPs for the disc of L2-L3 are given. The median and ranges for all recorded IDPs measured in both L2-L3 and L4-L5 during different activities are summarized in Table 1. No significant differences were found between lying during surgery (0.43 MPa, range: 0.41-0.43 MPa) and nocturnal sleeping (0.43 MPa, range: 0.38-0.47 MPa). Relaxed standing yielded an IDP value of approximately 0.70 MPa (range: 0.60-0.91 MPa). In contrast to *static activities*, *dynamic activities* caused a substantially higher IDP. The highest values were measured for standing up with ~3.73 MPa (range: 3.34-4.50 MPa) followed by lying down with ~2.25 MPa (range: 2.20–2.31 MPa) and walking with ~1.60 MPa (range: 1.34-3.58 MPa).

**Table 1:** Intradiscal Pressures (MPa) for different *static* and *dynamic activities*.

	**L2-L3**			**L4-L5**		
	**median**	**range**+	**range -**	**median**	**range**+	**range -**
lying intra-op	0.43	0.00	0.02	0.53	0.00	0.02
lying post-op	0.73	0.19	0.09	0.86	0.16	0.05
sleeping	0.43	0.04	0.05	0.51	0.03	0.02
standing	0.70	0.21	0.10	0.73	0.32	0.09
lying down	2.25	0.06	0.06	2.38	0.08	0.07
standing up	3.73	0.77	0.39	4.78	0.12	1.85
walking	1.60	1.98	0.26	1.48	1.74	0.28

**Figure 5:**
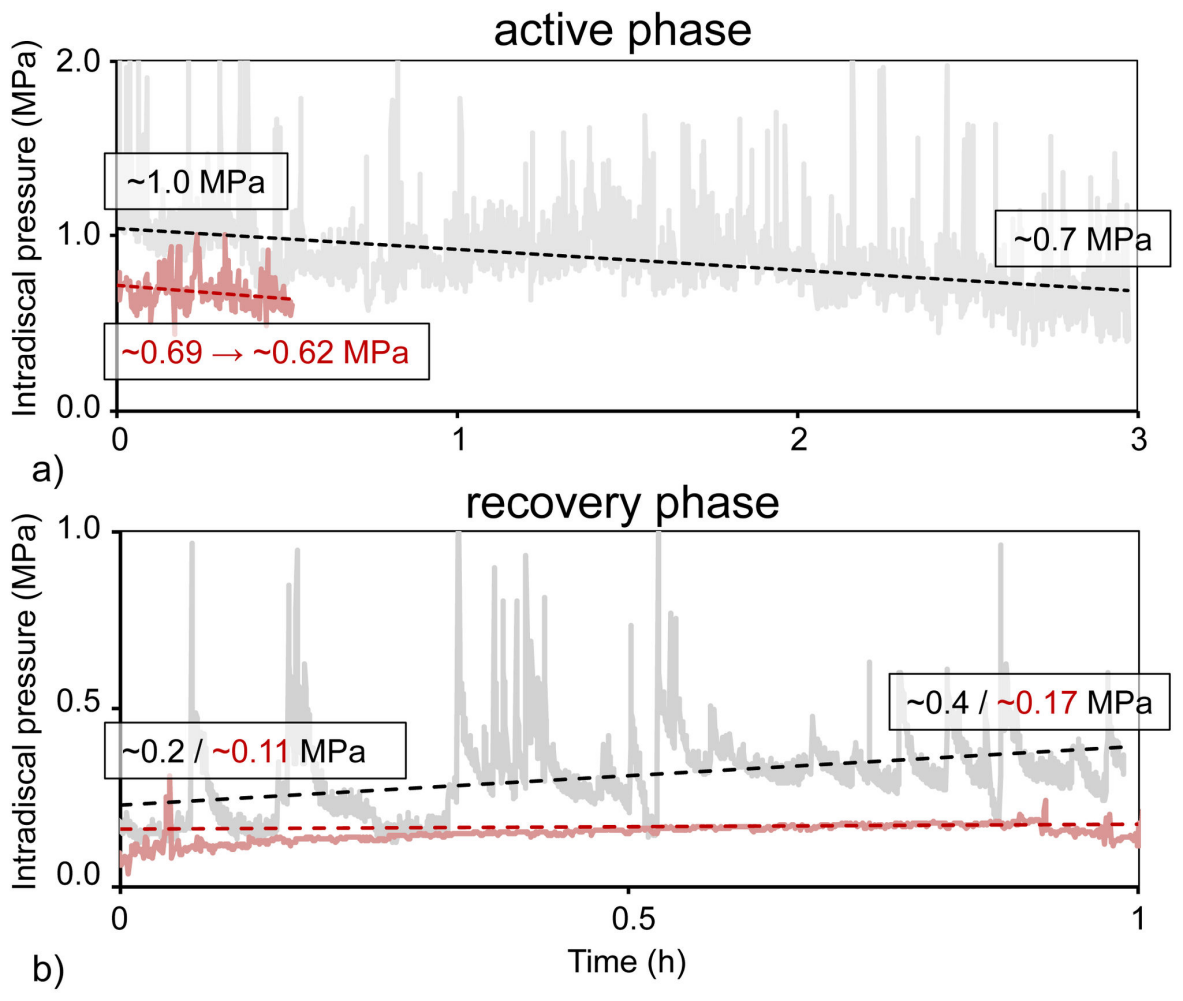
Concise plottings of continuous pressure records. a) Representative course of IDP throughout three hours of activity and b) one hour of rest for the sheep (gray) and the human (red). Due to the lack of continuous recordings of the human IDP profile during the day, only a trend in the course of the pressure is shown.

A continuous three hours of activity decreased the IDP from ~1.0 to ~0.7 MPa (Figure 5a). In contrast, a continuous lying phase of one hour induced an IDP increase from ~0.2 to ~0.4 MPa (Figure 5b). The rate of pressure rise during rest was, with 0.2 MPa/hour, twice as high as the loss in pressure during the activity phase, of 0.1 MPa/hour.

### Laboratory experiment

**Figure 6:**
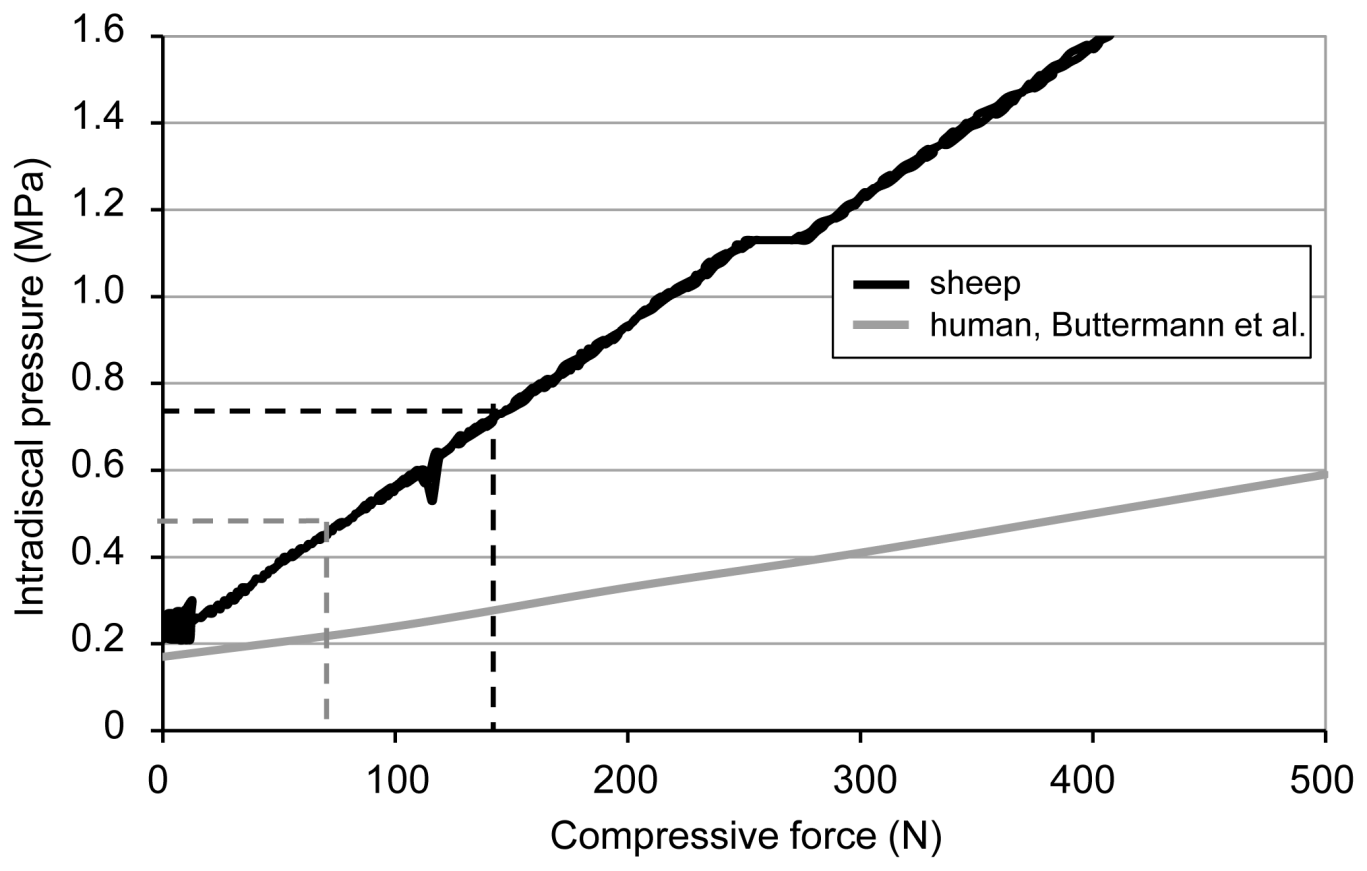
*Ex vivo* correlation between axial compressive force and IDP. The linear correlation between axial compressive force and IDP in the explanted ovine motion segments (L2-L3 shown) is markedly steeper than in humans [30]. The mean *in vivo* values of 0.75 and 0.5 MPa for *active phases* and *rest*, respectively, corresponded to axial compressive forces of 130 and 58 N.

After sacrificing the animal, a linear correlation between applied force and measured IDP was found in the axial compression test on the explanted segments (Figure 6). IDP values of 0.50 and 0.75 MPa corresponded to axial forces of 58 and 130 N, respectively.

## Discussion

The sheep is one of the most frequently used large animals for experimental spinal research. Selected for age, breed and sex, sheep normally show a great homogeneity [14]. However, whether the mechanical micro-environment of the disc *in vivo*, which is crucial for any kind of cellular process, justifies the use of sheep as a valid translational model for human disc regeneration strategies has not yet been investigated.

Comparing current results on ovine IDPs with existing human data [24–26] the intervertebral disc of the sheep appears to be consistently subjected to higher pressures than the human disc (Figure 4). This becomes particularly evident for *static activities*, like lying or sleeping, in which the ovine disc experiences approximately four times the loads measured in the human disc. Similarly, the loads of ovine discs during *dynamic activities*, such as getting up and walking around, are at least two to three times higher than in the human disc. Results of the current study on sheep, e.g. the IDP for standing being 0.70 MPa (L2-L3, range: 0.60‑0.91 MPa), are consistent with an earlier *in vivo* study investigating the nucleus pressure on mature runt cows, which detected values of approximately 0.80 +/‑0.24 MPa [30]. Lowest IDPs for lying were measured during surgery, which might be caused by the muscle relaxant effect of narcotic drugs. In contrast, highest IDPs for lying were measured directly after surgery, indicating that the anesthetic effects of medication had ceased, and disc fluid pressurization was fully regained after a long rest period.

The finding of comparably high ovine IDPs is in contrast to the conjecture that, due to the horizontally aligned spine of these animals, intradiscal loads should be less than in the upright positioned spine of man. In contrast to this assumption and in agreement with the present results, computational approaches approximating the load exposure acting on the spine of quadrupeds inferred that spinal structures in animals might be affected by even larger loads than in humans. The calculated gravitational bending moment in the static condition of the quadrupedal spine was found to be at least a factor of four times larger than the maximum possible permitted degree of physiological flexion and extension [13,31]. Without counterbalancing the gravitational force caused by the abdominal viscera, the quadrupedal spine would exhibit a lordotic posture. However, this is not the case as the quadrupedal thoracolumbar spine is adjusted to a kyphotic position. Consequently, there must be a redirection of loads at the horizontal spine of animals from bending to axial compression, resulting in a similar effective direction of force to that resting on the erect human spine. Similar to a suspension bridge between its pillars the thoracolumbar spine of sheep seems to be braced between the front and hind limbs through passive support mechanisms to avoid tensile stresses induced by unphysiological bending moments to the spine. Support for this hypothesis includes the quite long and strongly pronounced bony spinous processes of the ovine thoracolumbar spine [14]. Here, similar to the supporting ropes at a suspension bridge, the autochthonous back muscles as well as a variety of ligaments attach, thereby interlocking the spine.

Although the general posture of the body is the same for standing and walking, the ovine IDP for walking was approximately twice as high as the ovine IDP for relaxed standing. In contrast, human IDPs for both activities do not markedly differ [24,25]. In general, triaxial motion patterns (flexion-extension, lateral bending and rotation) were found to exist both in human and in quadrupedal motion segments during locomotion [32,33]. However, looking at a quadruped when galloping, with sequential series of suspension with the back and front legs coming together, one might get the impression that quadrupedal discs are subjected to higher bending moments during locomotion than human ones with predominantly axial compression. As flexion mainly stresses the disc and ligaments, spinal muscles attempt to absorb bending moments and convert them into stabilizing axial compressive loads [34,35]. For the sake of spinal stability during locomotion in quadrupeds, therefore, a higher muscular activity might be needed to prevent sagittal displacements of the spine, thus resulting in comparatively high intradiscal loads.

In addition to these theoretical and sophisticated interpretations, increased bone mineral density (BMD) in the vertebrae of quadrupeds and the craniocaudal orientation of its trabeculae (Wolff’s law [31]), both of which directly correlate to the magnitude and direction of the mechanical stresses present, underpin the current result that the quadrupedal spine experiences comparably high mechanical loads.

The axial compressive forces corresponding to the *in vivo* measured pressure values for *active phases* and *rest phases* were 130 and 58 N. This is in good agreement with a previous *in vivo* study investigating axial lumbar spinal loads on sheep [36] where the average value for standing was approximately 161 N. Compared to the axial forces derived from *in vivo* IDP measurements on humans with 400-600 N for standing and 100 N for lying, ovine values turned out to be much lower [37,38]. The slope of the regression curve between IDP and axial compressive force is markedly steeper for the ovine disc compared to results on human motion segments published by Buttermann et al. (Figure 6). Minor increases in axial compressive forces are thus causing a disproportionately higher increase in the ovine IDP. This result endorses the assertion that the overall smaller cross sectional area and height of the ovine disc is mainly responsible for the extent of variance in the biomechanical behavior of the ovine disc in comparison to humans. However, comparative numerical simulations [39] emphasized that next to the major differences in geometry between sheep and humans, divergences in material properties of the disc are also responsible for the differences in magnitude of IDP and axial compressive stiffness. While these differences may represent evolutionary adaptations to the requirements of bipedal or quadrupedal gait, the overall mechanical response of the disc still seems to be preserved across both species. This might be reflected in the temporal patterns of IDP between sheep and humans with similar trends of pressure decreases during activity phases and pressure increases during rest (Figure 5).

Despite the limitation that the study involves only one sheep, it provides a fundamental experimental attempt to gain insight into the loads acting on the ovine spinal column and to serve as a preliminary work for further investigations. Strong disparities in results between different animals of the same breed with healthy discs are not expected as there is evidence in the literature to suggest that in contrast to degenerated motion segments, inter-individual IDP differences in non-degenerated motion segments are only slight [40]. In the present study the age of the sheep was 3 years. In this age group disc degeneration is not usually found, as degenerative changes within the ovine intervertebral disc seem to develop at around 6 years of age [41]. Repeating the measurements of each single activity at various time points should ensure reproducibility of data even though only a single sheep was used.

A biomechanical limitation of the technique of IDP measurement might consist in the annulus defect. Disc puncture is commonly used to reliably initiate degeneration in several animal models [42–44]. Combined with a gradual catabolic reaction of living cells, the IDP progressively decreases *in vivo*. Lasting 24 hours, the time frame of IDP measurement in the current study is comparably short and degenerative changes due to disc puncture therefore are not expected. Additionally, a 4 mm cut in the annulus was shown not to significantly impair axial compressive stiffness and IDP of the ovine intervertebral disc [45,46].

The *in vitro* tests were performed under single axial compressive forces. As mentioned above, axial compression is the primary component of load on spinal motion segments in various daily activities, but combinations of sagittal, lateral and axial moments, and shear forces are also present to various degrees in many tasks. Forces derived from simplified laboratory experiments may therefore never be transferred in their whole extent to the *in vivo* situation.

This study offers a valuable insight into the mechanical stresses within the ovine intervertebral disc. It is the first study to focus on the *in vivo* measurement of the ovine intradiscal pressure. At present, the consequences of the pressure differences in IDP between the ovine and the human intervertebral disc remain unknown. However, future animal *in vivo* studies using the ovine intervertebral disc should bear these differences in mind and inferences from the model to the human field should be approached cautiously. As risks and prospects of success of new tissue engineering approaches cannot be mimicked *in vitro*, the sheep is still deemed useful as an animal model for preclinical research questions. To more deeply characterize the sheep as an animal model for intervertebral disc research, future studies should include a larger sample size, which would allow for statistical analysis to be conducted.
